# Photoplethysmography and ultrasonic-measurement-integrated simulation to clarify the relation between two-dimensional unsteady blood flow field and forward and backward waves in a carotid artery

**DOI:** 10.1007/s11517-016-1543-4

**Published:** 2016-07-27

**Authors:** Shusaku Sone, Toshiyuki Hayase, Kenichi Funamoto, Atsushi Shirai

**Affiliations:** 1 Graduate School of Biomedical Engineering, Tohoku University, Sendai, 980-8579 Japan; 2 EKG Technology Lab, Inc., Funabashi, 273-0864 Japan; 3 Institute of Fluid Science, Tohoku University, Sendai, 980-8577 Japan; 4 Frontier Research Institute for Interdisciplinary Sciences, Tohoku University, Sendai, 980-8578 Japan

**Keywords:** Hemodynamics, Pulse wave, Wave intensity, Wall shear stress, Carotid artery

## Abstract

Understanding the spatiotemporal change in hemodynamics is essential for the basic research of atherosclerosis. The objective of this study was to establish a methodology to clarify the relation between a two-dimensional (2D) unsteady blood flow field and forward and backward propagating waves in a carotid artery. This study utilized photoplethysmography (PPG) for blood pressure measurement and two-dimensional ultrasonic-measurement-integrated (2D-UMI) simulation for flow field analysis. The validity of the methodology was confirmed in an experiment for a carotid artery of a healthy volunteer. Synchronization between the pressure measurement and flow field analysis was achieved with an error of <10 ms. A 2D unsteady blood flow field in the carotid artery was characterized in relation to forward and backward waves. 2D-UMI simulation reproduced the flow field in which the wall shear stress takes a maximum at the time of the backward wave superiority in the systolic phase, whereas 2D ordinary simulation failed to reproduce this feature because of poor reproducibility of velocity distribution. In conclusion, the proposed methodology using PPG and 2D-UMI simulation was shown to be a potential tool to clarify the relation between 2D unsteady blood flow field and the forward and backward waves in a carotid artery.

## Introduction

Prevention and early detection of arteriosclerosis are important in all circulatory diseases [29]. Typical diagnostic methods of arteriosclerosis evaluate the systolic and diastolic blood pressure [2], the pulse wave velocity (PWV) [30], and the augmentation index (AI) [18], or the morphology of the cross section of a blood vessel, such as atheroma and calcification [19], and intima-media thickness using an ultrasonic diagnostic apparatus [6, 20].

Progression of arteriosclerosis is thought to be closely related to hydrodynamics. Mechanical stresses on the blood vessel wall, namely extension tension, transmural pressure, and wall shear stress (WSS), influence the function of endothelial cells and finally the entire blood vessel. Matsumoto et al. [24, 25] measured the change in the aorta of rats in a hypertensive state and found that wall thickness and outer diameter increase but that the inner diameters remain constant. Kamiya [16] and Langille et al. [21] reported that according to the change in flow rate, the internal blood vessel diameter changes so as to keep the WSS constant. Based on the fact that change in the blood vessel does not occur when endothelial cells are peeled off in advance, this reaction depends on endothelial cells [21]. It is known that the part of the blood vessel near the carotid sinus is a common site of atheroma. Caro et al. [3] reported that the non-planar curvature and branching of arteries average WSS acting on the vessel wall preventing the progress of blood vessel lesion. Long et al. [22] conducted magnetic resonance imaging measurement and a computational fluid dynamics simulation for the flow near the carotid sinus, showing that swirling and separation occurred in a non-planar branch near the carotid sinus. It has also been pointed out that atherosclerosis possibly develops in a region of low WSS where mass transport between the blood vessel and the blood decreases [4, 23].

The time-dependent property of hemodynamics is thought to be important in relation to arteriosclerosis since the entity of the blood flow in the blood vessel is properly described as a wave of pressure and velocity caused by the heartbeat. The relative phase of pressure and velocity, or equivalently the pressure and WSS acting on endothelial cells on the vessel surface, is different between the forward propagating wave from the heart and the backward propagating wave generated by the reflection from peripheral sites. It is, therefore, important in the analysis of hemodynamics to consider the relative phase between the blood pressure and the WSS acting on blood vessel wall in order to reveal the mechanisms of circulatory diseases and develop an effective diagnostic method. However, there have been few reports in the literature from such a viewpoint.

Parker et al. [31, 32] proposed wave intensity (WI) as a diagnostic parameter that can identify the forward and backward waves. The parameter WI, defined as the product of derivatives of the blood pressure and the cross-sectional average inflow velocity, has a positive value in forward waves and a negative value in backward ones. As a method to measure the blood pressure and the flow simultaneously, direct measurement by a catheter [12, 13] is invasive. Measurement using ultrasound equipment is a noninvasive method, in which the blood pressure is evaluated by blood vessel diameter and blood flow is evaluated by Doppler velocity [27, 28]. The authors have proposed a simultaneous analysis system using photoplethysmography (PPG) for pressure and two-dimensional ultrasonic-measurement-integrated (2D-UMI) simulation for two-dimensional (2D) flow analysis [37]. In 2D-UMI simulation, the feedback signal to compensate the differences between measured and computed Doppler velocities makes the computational result to converge to that of the real blood flow [9, 10]. However, simultaneity was not fully assured in the former system [37] between blood pressure measurement by PPG and blood velocity obtained by 2D-UMI simulation, and therefore, the results of analysis were only qualitative.

The objective of this study was to establish a methodology to clarify the relation between a 2D unsteady blood flow field and forward and backward waves in a carotid artery. As a key issue of this study, synchronization between pressure measurement by PPG and flow analysis by 2D-UMI simulation was developed. The validity of the methodology was confirmed in an experiment on a carotid artery of a healthy volunteer. A 2D unsteady blood flow field in the carotid artery was investigated in relation to forward and backward waves.

## Methods

In this section, the methodology for simultaneous pressure measurement and 2D flow field analysis is first explained. After showing the structure of the whole methodology, details on blood pressure measurement by a PPG sensor, 2D flow field analysis by 2D-UMI simulation, and time phase synchronization between pressure measurement and 2D flow field analysis are described. Then, the method for a confirmation experiment is presented.

### Methodology for simultaneous pressure measurement and 2D flow field analysis

**Fig. 1:**
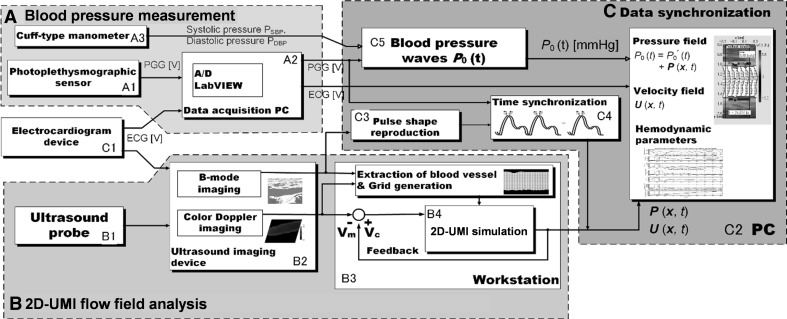
Block diagram of the simultaneous analysis system of blood pressure and flow using photoplethysmography and ultrasonic-measurement-integrated simulation

A block diagram of the developed analysis system is shown in Fig. 1. The system consists of a blood pressure measurement component (A), a 2D-UMI flow field analysis component (B), and a data synchronization component (C). In the figure, the blood pressure measurement component (A) consists of a PPG sensor (A1), a data acquisition PC (Precision M6700, OS Windows7, Dell, USA) with an A/D converter (NI 9215, 16 bit, 200 Hz, National Instruments, USA) (A2), and a cuff-type manometer (HEM-9000AI, Omron, Japan) (A3). An analog signal from the PPG sensor (A1) is transformed into a digital PPG pulse signal (V) in the A/D convertor. The 2D-UMI flow field analysis component (B) consists of a linear ultrasound probe (10L, center frequency 5 MHz, pulse repetition frequency 4.4 kHz, GE Healthcare Japan, Japan), (B1), ultrasound diagnostic imaging equipment (LOGIQ 7, GE Healthcare Japan, Japan) (B2), and a workstation (Altix XE500, CPU Xeon 2.93 GHz 8 CPU, memory 48 Gbyte, OS SuSE Linux Enterprise Server 10 SP2, Compiler Intel Fortran Compiler 11, Intel C/C ++ Compiler 11, SGI Japan, Japan) (B3). Blood vessel shape and blood flow Doppler signal are acquired by B-mode and color Doppler imaging mode measurement with the ultrasound diagnostic imaging equipment (B2), and the blood flow field is obtained by 2D-UMI simulation (B4) in the workstation (B3). The data synchronization component (C) consists of an electrocardiogram (ECG) (C1) and an analysis PC (Surface Pro2, OS Windows 8.1, Microsoft, Inc., USA) (C2). A subject’s ECG waveform is measured by using the ECG sensor (C1). The measured ECG signal is filtered by a notch filter (−90 dB at 50 Hz, bandwidth 10 Hz), a high-pass filter (FIR filter, 250th order, stop-band 2 Hz, pass-band 5 Hz, sampling 200 Hz), and a low-pass filter (FIR filter, 200th order, stop-band 30 Hz, pass-band 20 Hz, sampling 200 Hz) to remove power-supply noise, baseline drift, and myoelectric noise, respectively. The time delay of the signal caused by the filtering is properly compensated. The filtered ECG signal is fed to the blood pressure measurement component (A) and the 2D-UMI flow field analysis component (B) to roughly synchronize the time phases. The data synchronization component (C) also has a function (C3) to extract the blood vessel shapes from the B-mode image data and to obtain a pulse wave from the blood vessel areas. This pulse wave defines the image pulse measured in pixels. Exact time synchronization between blood pressure measurement in component A and blood flow field analysis in component B is performed by adjusting the time phase of the image pulse waveform obtained in C3 to that of the PPG waveform obtained in A2. The blood pressure pulse signal $P_{0} (t)\,({\text{mmHg}})$ is determined in C5 by applying systolic and diastolic pressure values obtained in A3 to maximum and minimum values of the PPG signal acquired in A2. Finally, exactly synchronized blood pressure change and 2D unsteady flow field in a blood vessel are acquired. They are used to calculate hemodynamic parameters such as PWV, pulse transit time (PTT), AI, and WI. Data processing in component C is performed by MATLAB (R2013a MathWorks, Inc. USA) on the analysis PC (C2).

#### Blood pressure measurement

Let us now describe the principle of blood pressure measurement based on blood vessel volume change used in both the PPG pulse in A2 and the image pulse in C3. Sugawara et al. [38] reported the linear relationship between the blood vessel diameter and intra-arterial pressure in a carotid artery based on pressure measurement by a catheter and blood vessel diameter measurement by ultrasonic equipment. Thus, it is possible to identify sequential blood pressure from change in the lumen size by applying systolic and diastolic blood pressure values measured in advance to their maximum and minimum values.

PPG measurement (A1) acquires a change in the volume of blood, particularly hemoglobin, in a blood vessel due to the change in lumen size. In the present reflection-type PPG sensor, a light source and a photodetector are positioned along a blood vessel axis. Light emitted from the light source is dispersed, absorbed, and reflected by tissues and blood, and reflected light is measured by the photodetector. In this study, this method was applied to measure the change in blood vessel volume during pulsation. It is noted that the transmission type [40] is commonly used in blood pressure and pulse measurement on the fingertip. The theoretical background of PPG measurement is briefly explained below. Hemoglobin, the main component of red blood cells, is dielectric with frequency-dispersive behavior. By Beer–Lambert’s law [40], light strength *I* changes against distance $\ell$ as follows:

1 $$I = |I_{0} |e^{ - \in (\lambda )C\ell }$$

where $\left| {I_{0} } \right|$ is the incident strength of the light, $\in (\lambda )\,({\text{L/mol}}\,{\text{cm}})$ is the molar absorption coefficient, *λ* is the wave length, and *C* (mol/L) is the molar concentration of the hemoglobin. The present PPG sensor uses the wavelength of incident light *λ* = 940 nm [40]. The molar concentration *C* in Eq. (1) is considered to be constant in the case of a common carotid artery with a diameter of approximately 5 mm since Barbee and Cokelet [1] showed that the molar concentration is independent of flow speed, vessel diameter, and stirring for blood vessels with a diameter of over 128 μm. Feng et al. [7] reported that the average photon migration path in the tissue was banana-shaped when a light source and a photodetector were lined up in a series. A light source and a photodetector in the sensor are located along a common carotid artery with 8-mm interval which was determined by trial and error.

Image pulse measurement (C3) is performed by the ultrasound diagnostic imaging equipment to exactly adjust the time phase of ultrasound measurement to that of PPG measurement. A 2D major axis section of the blood vessel is measured in the B-mode. To extract the blood vessel area, the B-mode images are binarized with a suitable threshold and classified as blood vessels and other tissues by using a watershed segmentation algorithm [26]. The blood vessel area is then computed in pixel units. By performing this operation under the sampling frequency of ultrasound measurement, the pulse wave is obtained from the time change in the cross-sectional area of the blood vessel.

#### 2D-UMI flow field analysis

In the 2D-UMI simulation in B4 (Fig. 1), a 2D blood vessel shape is first extracted from the ultrasound measurement data in B2, and the computational domain is defined by an orthogonal grid system. 2D-UMI simulation is then performed using Doppler velocity measurement data [17]. For the sake of simplicity, 2D flow on a cross section along the axis of a rigid blood vessel is analyzed ignoring the three-dimensional characteristics and deformation of the blood vessel during pulsation.

The governing equations are the 2D Navier–Stokes equations and pressure equation:
where  is the velocity vector, *p* is the pressure, *t* is the time, *ρ* is the density, *μ* is the viscosity, and  is the artificial body force defined as the feedback signal of UMI simulation.where *K*_v_^*^ is the feedback gain (non-dimensional), *U* is the characteristic velocity, *L* is the characteristic length, and  is the unit vector along the ultrasound beam. *V*_c_ and *V*_m_ are Doppler velocities, projection of the velocity vector in the direction of the ultrasonic beam, of the computation and measurement, respectively. In this method, the numerical result converges to the actual blood flow field by the effect of the feedback signal defined in the feedback domain in the computational domain. Procedures for the extraction of blood vessel shape and generation of the computational grid follow Ref. [17]. The orthogonal *x*–*y* coordinate system is defined with the *x*-axis along the blood vessel axis in the downstream direction and the *y*-axis in the depth direction. An orthogonal equidistant staggered grid is defined based on clinical data with grid spacing identical to the spatial resolutions of the ultrasonic measurement. The blood vessel shape is rotated so that the longitudinal direction of the blood vessel agrees with the *x*-axis. Governing equations are discretized with the finite volume method and solved with an algorithm similar to the SIMPLER method [34]. The convective terms are discretized with the reformulated QUICK scheme [14], and the time derivative terms are discretized with a second-order implicit scheme. Linear algebraic equations are solved using the MSI scheme for pentadiagonal matrices [36]. According to Kato et al. [17], the feedback domain was set in the computational domain. As a boundary condition, a parabolic velocity profile with a mean inlet velocity estimated by the golden section search method is applied at the upstream boundary. Free-flow and no-slip conditions are applied at the downstream boundary and on the vessel walls. The effect of the measurement errors on the analysis was properly compensated [11].

#### Synchronization between blood pressure measurement and blood flow analysis

For the synchronization between PPG blood pressure measurement and 2D-UMI flow field analysis (Fig. 1, C4), the system clock of the data acquisition PC (A2) for PPG measurement and that of the ultrasound imaging device (B2) for blood flow analysis were synchronized. The system clock of the ultrasound imaging equipment (B2) was adjusted by a 1-ms unit to minimize the square mean difference for six cardiac cycles between peak times of the PPG pulse waveforms acquired in A2 and those of the image pulse waveforms acquired in C3.

### Confirmation experiment

An experiment was performed to confirm the validity of the proposed methodology. The objective was the blood flow in the left carotid artery of a healthy 23-year-old male volunteer. This study was approved by the ethics committee of the Institute of Fluid Science, Tohoku University, and was performed with informed consent of the volunteer. First, the volunteer’s systolic and diastolic blood pressures were measured with a cuff-type manometer. Next, ECG sensors were attached to both wrists and the left ankle of the volunteer, the PPG sensor was held on the common carotid artery by tape, and measurement of the ECG and PPG signals was started. During measurement of ECG and PPG, the ultrasound linear probe was held over the left common carotid artery and ultrasound color Doppler images were acquired for seven cardiac cycles. After the ultrasound measurement, the volunteer raised his hand to enable recording of characteristic signals in ECG and ultrasound measurements, which were used to determine the correspondence of wave patterns of blood pressure and Doppler velocity in later analysis. The above-mentioned measurement was repeated three to five times at intervals of almost 2 min. After these measurements, systolic and diastolic blood pressures were measured again by the cuff-type manometer. Reference values for the systolic and diastolic blood pressures were determined by averaging the results of the cuff-type manometer obtained before and after the PPG and ultrasound measurements.

The sampling frequencies of the color Doppler and B-mode measurements were both 34.394 Hz. The spatial resolutions of color Doppler measurement were ∆*x* = 203 μm and ∆*y* = 194 μm, corresponding to the numbers of measurement points, *N*_x_ × *N*_y_ = 71 × 91. Those of the B-mode measurement were ∆*x* = 307 μm and ∆*y* = 97 μm, and *N*_x_ × *N*_y_ = 128 × 415.

The calculation condition of 2D-UMI simulation is next described. The blood was assumed to be Newtonian fluid with density *ρ* and viscosity *μ* of 1.0 × 10^3^ kg/m^3^ and 4.0 × 10^−3^ Pa s, respectively. The number of grid points *N*_x_ × *N*_y_ was 36 × 86 with grid spacing of ∆*x* = 163 μm and ∆*y* = 173 μm, respectively. The feedback domain was defined from 1/36 to 29/36 of the computational domain from the upstream boundary. It should be noted that the computational domain was reduced through extraction of a rectangular domain from an original rhombus domain of color Doppler measurement (see Fig. 3a). The computational time step 29.075 ms (34.394 Hz) agreed with that of the ultrasound measurement. The convergence criteria for non-dimensional residuals in the flow analysis and the estimation of the cross-sectional average inlet velocity were set as 1.0 × 10^−2^ [–] and 1.0 × 10^−3^ [–], respectively, by compromising computational time and accuracy. The characteristic length *L*, velocity *U*, and density *ρ* for non-dimensionalization were defined as a blood vessel diameter of 6.4 mm at the upstream boundary, an arbitrary value of 0.1 m/s, and a blood density of 1.0 × 10^3^ kg/m^3^, respectively. Feedback gain of the 2D-UMI simulation was set to *K*_v_^*^ = 500, referring to a previous study [17]. For the purpose of comparison, a 2D ordinary (2D-O) simulation with the null feedback gain, *K*_v_^*^ = 0, was also performed. Typical computational time for 2D-UMI and 2D-O simulations performed in the workstation was 14 s for one time step.

Evaluation of blood pressure measurement was performed by comparing the waveform of PPG with that of the image pulse. The waveform of PPG was normalized between 0 and 1 by using its minimum and maximum values in each measurement, and the waveform of the image pulse was scaled by the least-squares method so as to minimize the error with respect to the normalized PPG waveform. Typical computational time to obtain the image pulse was 70 s for one time step, while those of other tasks performed in the analysis PC were negligibly small.

To evaluate the blood flow field obtained in both 2D-O and 2D-UMI simulations, the error norm of Doppler velocity *e*(*t*) was defined as follows:

5 $$e\left( t \right) = \frac{1}{{N_{\text{e}} }}\sum\limits_{n} {\left| {V_{\text{c}} \left( {n,t} \right) - V_{\text{m}} \left( {n,t} \right)} \right|} /V_{\text{type}} ,$$

where *n* is the grid index, *N*_e_ is the total number of grid points in the feedback domain, and *V*_type_ is a typical blood flow velocity in a common carotid artery, 0.39 m/s [35].

The evaluation of time synchronization of pressure measurement and 2D flow field analysis is now explained. Based on the water hammer theory, the waveforms of flow and pressure in the systolic phase consist of only forward propagating waves, their rising edges being aligned at the same instant [31]. Therefore, the PU loop [31], namely a graph consisting of pressure *P* on the vertical axis and flow velocity *U* on the horizontal axis, in the systolic phase is a straight line when the blood pressure measurement and flow analysis are exactly synchronized, a convex curve for advanced pressure measurement, or a concave curve for delayed pressure measurement.

## Results

### Simultaneous pressure measurement and 2D flow field analysis

**Fig. 2:**
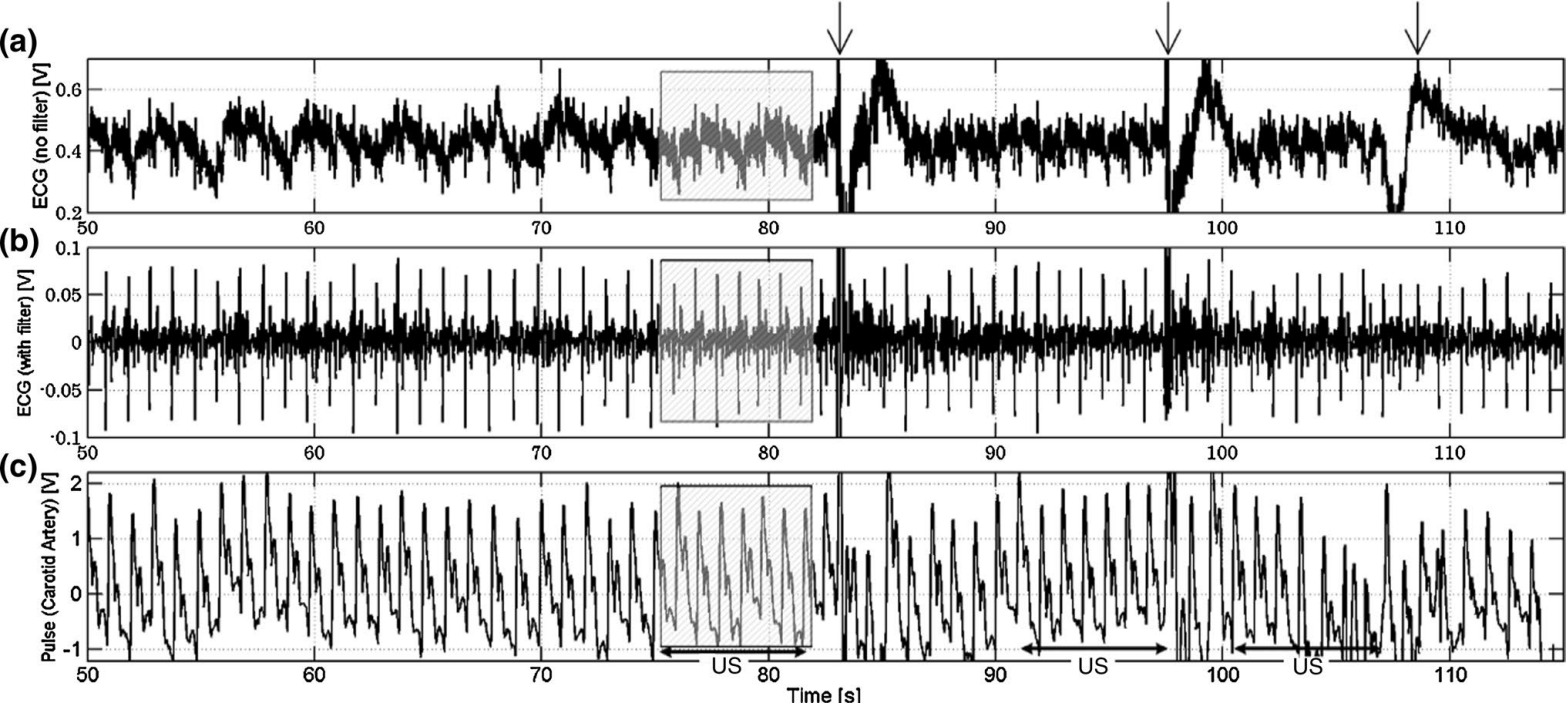
Measurement data: **a** raw ECG signal; **b** ECG signal after filter processing; **c** PPG signal in a *left* carotid artery. Frames shown as US indicate parts undergoing ultrasound measurement simultaneously. *Arrows* indicate disturbances of the waveform caused by raising of the volunteer’s hand to mark completion of measurements

Figure 2a–c shows raw ECG data, filtered ECG data, and raw PPG data, respectively. In Fig. 2a, disturbances of the waveform indicated by arrows were caused by raising of the volunteer’s hand to mark completion of measurements. The ultrasound measurement was taken in the parts shown as US in Fig. 2c. In the following, analysis was conducted for the data in the period from *t* = 75.275–81.990 s (shaded region in Fig. 2).

**Fig. 3:**
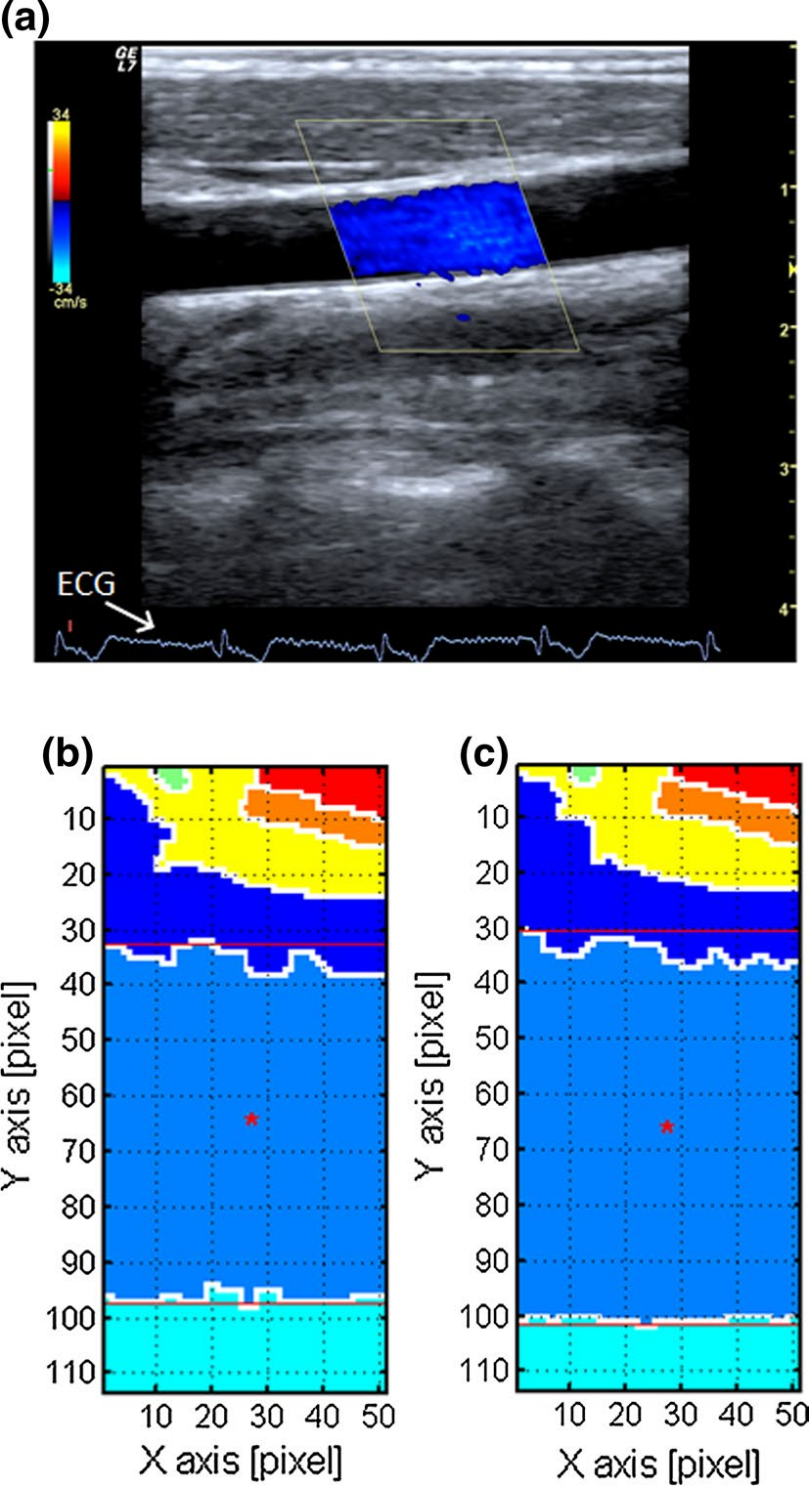
Objectives of analysis in a left carotid artery: **a** ultrasound measurement. ECG signal is shown in the lower part; **b** extracted image of the blood vessel in the diastolic phase (*t* = 78.764 s); **c** extracted image of the blood vessel in the systolic phase (*t* = 78.851 s)

An example of color Doppler image data is shown in Fig. 3a. The ECG signal in Fig. 3a (indicated by an arrow) shows the same signal as that in Fig. 2b, which was used for rough synchronization of the time phase. In Fig. 3b, c, domains from roughly 30 pixels to 100 pixels in the *y*-direction show the blood vessel area extracted by the watershed segmentation algorithm from the B-mode images corresponding to the time of the smallest cross-sectional area (*t* = 78.764 s) and the time of the largest one (*t* = 78.851 s), respectively.

**Fig. 4:**
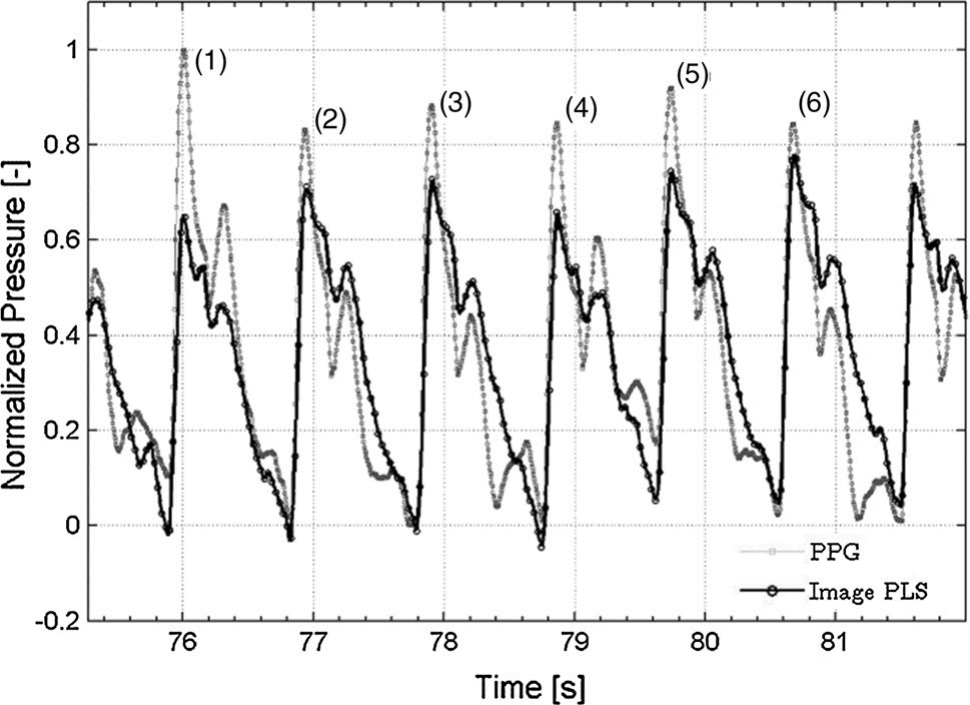
Normalized PPG pulse waveform and scaled image pulse waveform with least square error

Figure 4 shows the PPG signal in six heart beats in the above-mentioned period (thin line) and the corresponding image pulse signal acquired from the ultrasonic images (bold line). As already mentioned, the waveform of the PPG signal was normalized between 0 and 1 and that of the image pulse signal was scaled by the least-squares method with respect to the normalized PPG waveform. The time phase of the image pulse waveform was adjusted by a method using the peak times of both waveforms. Close examination of the waveforms shows that the first peaks of the image pulses are smaller than those of the PPG pulses, but subsequent values are larger.

**Fig. 5:**
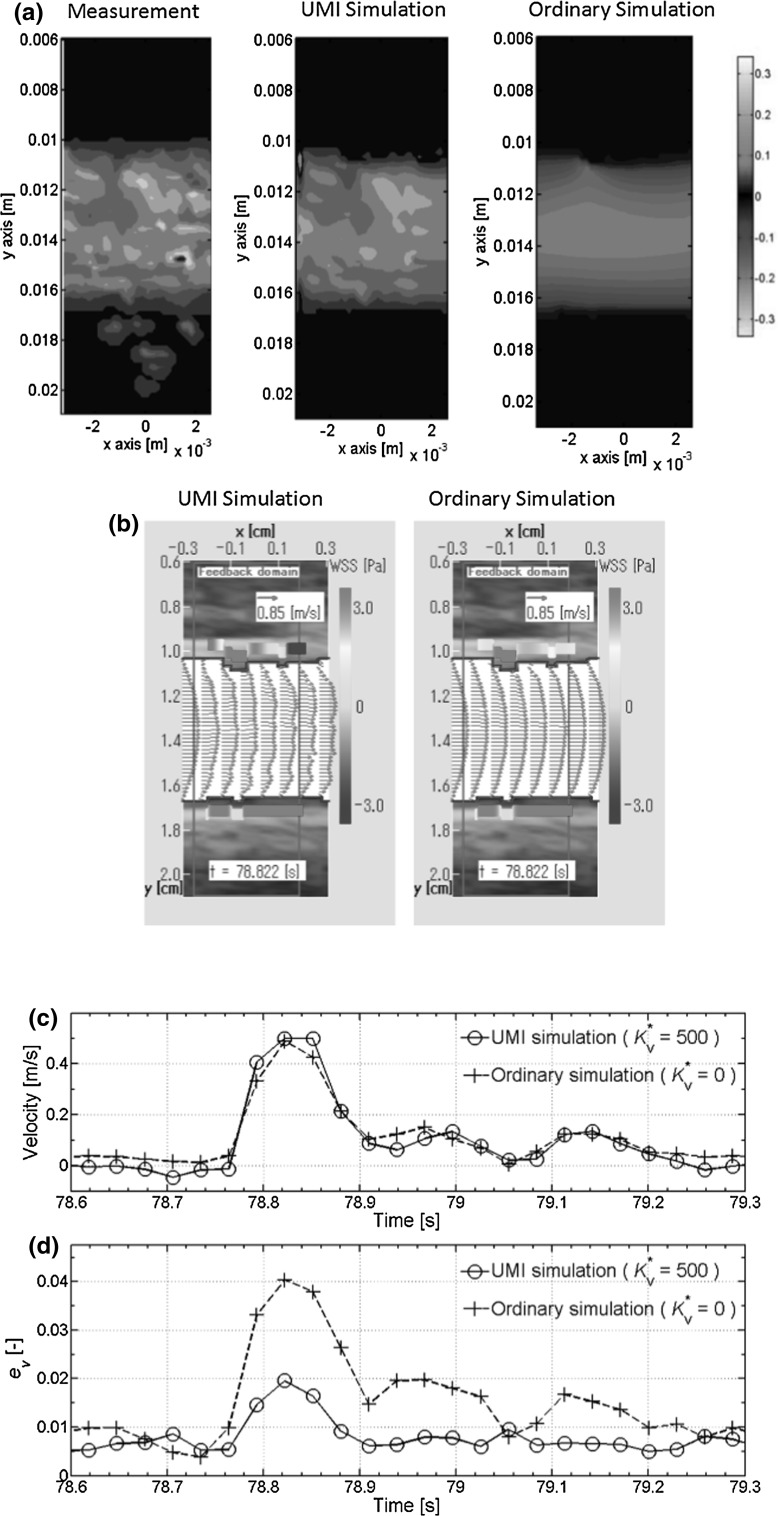
Results of 2D-UMI and 2D-O simulation: **a** measurement of Doppler velocity field in the systolic phase (*t* = 78.822 s) (*left*), the 2D-UMI simulation (*middle*), the 2D-O simulation (*right*); **b** velocity field calculated by the 2D-UMI simulation (*left*) and the 2D-O simulation (*right*); **c** cross-sectional average inflow velocity; **d** space-averaged error norm of Doppler velocity

The result of 2D-UMI simulation for the blood flow field is described below. The data used in feedback signal were the color Doppler data in the above-mentioned period. The left side in Fig. 5a shows the measurement data of Doppler velocity distribution at the peak of the systolic phase of the PPG pulses [(4) in Fig. 4]. The result of 2D-UMI simulation [middle in Fig. 5a] represents the Doppler velocity distribution similar to that of the measurement data, while the result of the 2D-O simulation [right-hand side figure in Fig. 5a] is different from that of the measurement. Figure 5b compares the velocity vector field between the 2D-UMI simulation (figure on the left) and the 2D-O simulation (figure on the right). Both the upstream boundary velocity profiles have the same parabolic shape, but the 2D-UMI simulation represents complex velocity distributions in the feedback domain, whereas the 2D-O simulation represents nearly parabolic distributions. Time variations in inflow velocity acquired by the golden section method in Fig. 5c show that the difference between the 2D-UMI and 2D-O simulations is small. However, Fig. 5d shows that the space-averaged error norm of the Doppler velocity of the 2D-UMI simulation is almost half that of the 2D-O simulation throughout the whole time.

**Fig. 6:**
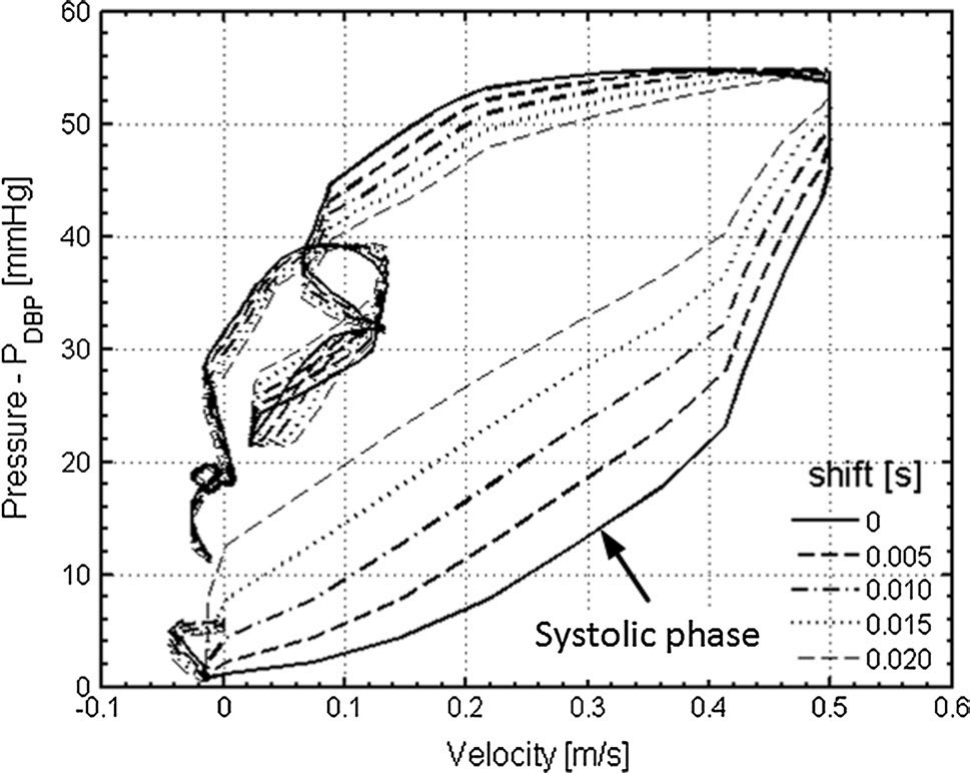
Trajectries of pressure and inflow velocity, namely PU loop, for one cardiac cycle shown in Fig. 5. Initial time of velocity variation is adjusted by 5 ms units

The synchronism for blood pressure measurement by PPG and blood flow analysis by 2D-UMI simulation is described below. The time needed to advance the measurement time of the ultrasonic measurement system was 29 ms to minimize the mean square error of the peak times between the PPG pulses and the image pulses from *t* = 75.835–81.435 s based on the method described in Sect. 2.1. The solid line in Fig. 6 represents a PU loop based on the blood pressure waveform obtained by the PPG sensor [(4) in Fig. 4] and the inflow velocity waveform obtained by 2D-UMI simulation (Fig. 5c). The PU loop in Fig. 6 has a concave shape in the systolic phase (arrowed). Therefore, 2D-UMI simulation was performed by delaying the time by 5 ms units, and a PU loop near a straight line was acquired when the time was advanced 5 or 10 ms (a dashed line or dot-dash line in Fig. 6). Further advancements of 15 ms and 20 ms result in a delay of velocity in early systole. From these results, the time error between the PPG pressure measurement and the 2D-UMI blood flow field analysis in the developed system is considered to be within 10 ms.

### Two-dimensional unsteady flow field in relation to forward and backward waves

**Fig. 7:**
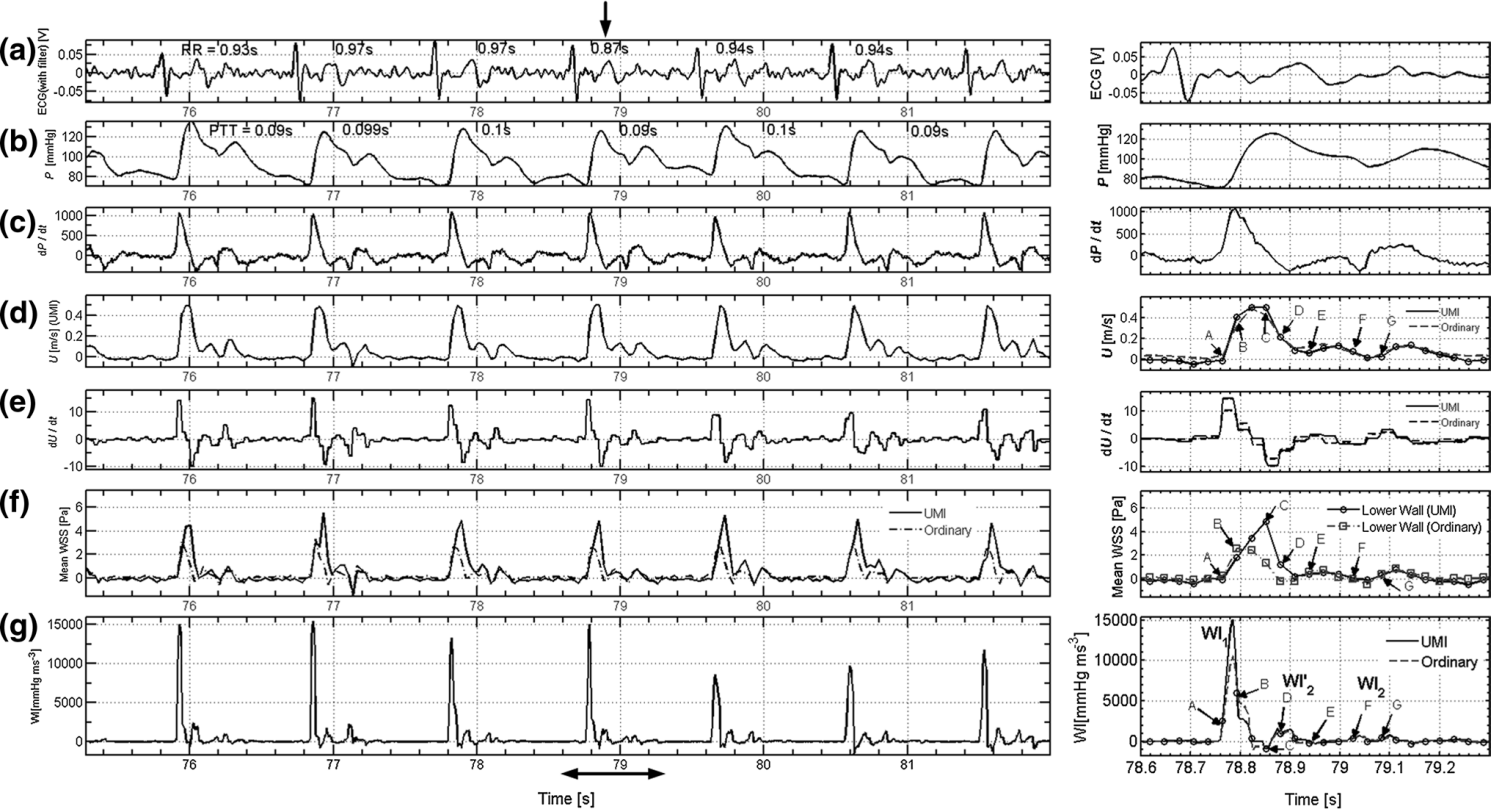
**a** ECG; **b** blood pressure pulse wave by PPG; **c** derivative of **b**; **d** inflow velocity; **e** derivative of **d**; **f** space-averaged WSS; **g** WI calculated by **c** and **e**. Figures on the *right* are enlarged figures of part of the fourth waveform indicated by a *double-headed* *arrow* in the figures on the *left*

The figures on the left in Fig. 7 show the results acquired by the system for (a) ECG, (b) blood pressure by PPG, (c) derivative of (b), (d) inflow velocities with 2D-UMI simulation, (e) derivatives of (d), (f) space-averaged WSS on lower wall by 2D-UMI simulation, (g) and WI, calculated by (c) and (e). Values of *R*–*R* interval time are shown in Fig. 7a, and those of pulse transit time (PTT) [5, 8], which represents the time from the ECG R wave peak to standing up pulse, are shown in Fig. 7b, respectively. *R* wave peaks were detected as the maximum value of the ECG signal in each cardiac cycle.

The figures on the right in Fig. 7 are enlarged figures of part of the fourth waveform in the figures on the left. In Fig. 7d–g, results of the 2D-O simulation are also plotted for comparison. A remarkable difference was observed between 2D-UMI and 2D-O simulations in Fig. 7f for space-averaged WSS in the lower wall in the period from *t* = 78.8–78.9 s near the systolic peak of velocity *U*. The 2D-UMI simulation has a maximum WSS at the time marked *C* with a value of almost 5 Pa, while the 2D-O simulation has a maximum WSS at the time marked B with a value of almost 2 Pa, less than half that of the 2D-UMI simulation. In Fig. 7d, the difference in the inflow velocity *U* is relatively small between 2D-UMI and 2D-O simulations.

**Fig. 8:**
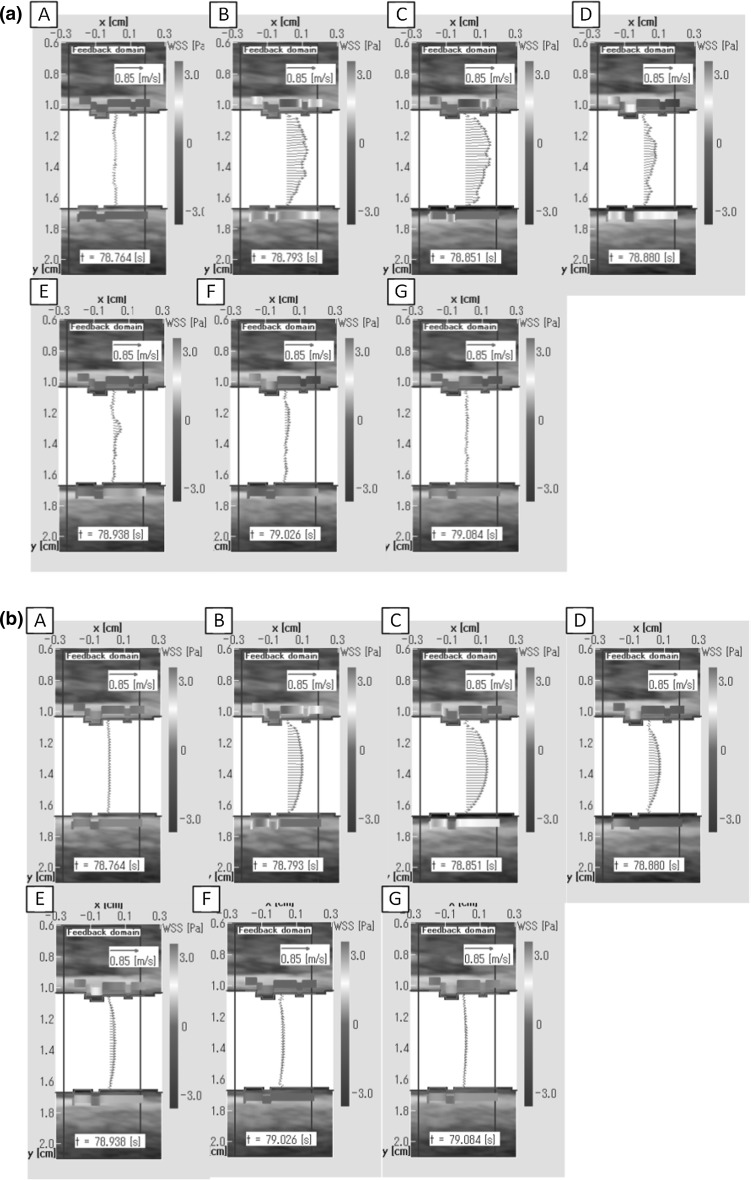
Distributions of velocity vector along the vessel diameter and WSS along *upper* and *lower* walls at instants corresponding to those of Fig. 7: **a** 2D-UMI simulation; **b** 2D-O simulation

Figure 8a, b shows a time series of velocity vector distributions in a cross section and WSS distribution along the upper and lower wall from the result of 2D-UMI and 2D-O simulations at the times (A)–(G) in Fig. 7, respectively.

## Discussion

In this study, a methodology was established to clarify the relation between a 2D unsteady blood flow field and forward and backward waves in a carotid artery by synchronizing blood pressure measurement with PPG and flow field analysis with 2D-UMI simulation.

Evidence for the validity of the pressure measurement is shown in Fig. 4, in which waveforms of PPG pulse and image pulse agree. The validity of 2D unsteady blood flow field analysis is shown in Fig. 5 in which error in Doppler velocity for 2D-UMI simulation is substantially reduced compared with that of 2D-O simulation. Synchronization between the pressure measurement and 2D flow field analysis is shown in Fig. 6, the time error between the PPG pressure measurement and the 2D-UMI blood flow field analysis being reasonably estimated to be within 10 ms. This indicates that the proposed methodology is sufficiently accurate to analyze pulse waves with frequency components up to 10 Hz or the like. The relations between a 2D unsteady blood flow field and forward and backward waves in a carotid artery are shown in Figs. 7 and 8. Time-varying distributions of velocity vector and WSS obtained by 2D-UMI simulation in Fig. 8a are clearly related to forward and backward waves through the sign of WI in Fig. 7g.

By checking signs of derivatives of pressure and velocity variations in Fig. 7c, e, and that of WI in Fig. 7g, the state of wave propagation in a blood vessel can be identified [31]. It is considered that there is wave propagation only with a forward propagating wave near the peak WI_1_ at *t* = 78.78 s between A and B in Fig. 7 when derivatives of pressure and velocity are both positive and WI is at a positive peak. It is also assumed that an absorption wave [33], which causes blood flow to stop, is produced near the peak WI_2_ at *t* = 79.03 s (F in Fig. 7) when derivatives of pressure and velocity at the end diastole are both negative and WI is at a positive peak after the *T* wave of ECG [33]. Niki et al. [27] reported that the peak of WI_2_ disappeared in case of mitral regurgitation. Furthermore, a negative peak *C* was observed at *t* = 78.85 s when the derivative of pressure was positive and that of flow velocity was negative. Another positive peak WI’_2_ was observed at *t* = 78.88 s (D in Fig. 7) when derivatives of pressure and flow were both negative, corresponding to the absorption wave.

In Fig. 7f, 2D-UMI simulation reproduced a flow field in which the WSS has a maximum at the time of the backward wave superiority in the systolic phase, whereas 2D-O simulation failed to reproduce this feature because of poor reproducibility of velocity distribution (Fig. 8b). In Fig. 8a for the 2D-UMI simulation, both the inflow velocity and the velocity gradient in the lower wall in (C) are larger than those in (B). In Fig. 8b for the 2D-O simulation, the inflow velocity in (C) is larger than that in (B), but the velocity gradient in (C) is smaller than that in (B). These results correspond to the difference in space-averaged WSS in 2D-UMI and 2D-O simulations shown in Fig. 7. Considering the results of WI, the WSS obtained in the 2D-UMI simulation was maximum when the backward propagating wave was dominant, whereas the WSS in the 2D-O simulation was maximum when forward propagating wave was dominant. This means that characteristic of the flow field obtained with the 2D-O simulation was physically different from that obtained with 2D-UMI simulation from the viewpoint of WSS variations. In the case of WI creating an absorption wave, it has been pointed out that there is a possibility of flow stoppage generating backward flow near a blood vessel wall [15, 39]. The results of (D) in the 2D-O simulation (Fig. 8b) show such flow stoppage. The corresponding result of 2D-UMI simulation (Fig. 8a) does not clearly show such flow, but a decrease in flow velocity was observed near the wall. From these results, it was suggested that the 2D-O simulation can possibly estimate the influence of wave propagation in a carotid artery on the blood vessel wall different from the actual one.

In previous studies [15], WI was analyzed based on the mean waveform using the ensemble average, but the developed system makes it possible to acquire sequential WI waveforms and corresponding flow fields during heartbeats. For example, the fifth heartbeat after a relatively short *R*–*R* interval (0.87 s) of ECG (arrow in Fig. 7a) results in relatively lower peaks in d*U*/d*t* and WI, the physical meaning of which remains for future study.

For precision in measurement of the image pulse, the vertical resolution of the B-mode image was 97 μm, corresponding to a blood vessel diameter of almost 70 pixels and a variation of almost 7 pixels; the time resolution was 29 ms. It is noted that the graph in Fig. 4 was plotted using cubic spline interpolation for their values. For measurement of PPG, its resolution was 0.15 mV, corresponding to an amplitude of almost 20,000 (3 V), and its time resolution was 5 ms. It is thought that information of higher resolution can be acquired by PPG rather than by image pulse. However, the problem of whether the change in blood vessel diameter correctly reflects that of the pressure has not been sufficiently verified, and thus, this is a problem for future study. It should also be noted that the blood pressure measured by PPG corresponds to the average pressure over the measurement domain in a blood vessel varying between 80 mm Hg and 120 mmHg during a cardiac cycle (see Fig. 7b), whereas the pressure obtained by 2D-UMI simulation is a relative pressure from this average value distributed over the domain with the amplitude on the order of 10 mmHg. Therefore, comparison between the blood pressure with PPG measurement and that of 2D-UMI simulation is not appropriate.

As limitations of this work, the blood pressure field and other hemodynamic parameters such as pulse wave velocity (PWV), pulse transit time (PTT), and augmentation index (AI) were not discussed because of space limitation. The validity of the present work should be evaluated for more cases in future study. This study also ignored the effect of three dimensionality of blood flow and that of elasticity of blood vessels, which should also be investigated.

## Conclusion

In this study, a method to clarify the relation between a 2D unsteady blood flow field and forward and backward waves in a carotid artery was investigated. Synchronization between pressure measurement with PPG and flow field analysis with 2D-UMI simulation was achieved with an error of less than 10 ms. As a result of a confirmation experiment on a carotid artery of a healthy volunteer, 2D unsteady blood flow field in the carotid artery was characterized in relation to forward and backward waves. 2D-UMI simulation reproduced the flow field in which the WSS had a maximum at the time of the backward wave superiority in the systolic phase, whereas 2D-O simulation failed to reproduce this feature because of poor reproducibility of velocity distribution. In conclusion, the present method is a potential tool to clarify the relation between 2D unsteady blood flow field and forward and backward waves in a carotid artery.
